# New Appliances and Things Medical

**Published:** 1906-02-24

**Authors:** 


### NEW APPLIANCES AND THINGS MEDICAL.

[We shall be glad to receive at our Office, 28 & 29 Southampton Street, Strand, London, W.C., from the manufacturers, specimens of all new preparations and appliances which may be brought out from time to time.]

### "FORMAWN."

(The Chemical Works, 26 Southwark Bridge Road, London, S.E.

Although the pathology of what we are pleased to call a "cold" is far from clear, the treatment of "catarrh" is a subject which interests the majority of mankind; and among life's minor ailments there are few which more baffle and perplex the general practitioner. The recently introduced "Formawn," or chlormethylmenthyl-ether, having the formula $C_{10}H_{19}OCh_2Cl$, is the latest anti-catarrhal agent. This substance, when brought in contact with damp air or warm water, splits up into formaldehyde, menthol, and hydrochloric acid. While the latter is taken up by the water, the two former are given off as vapour. For purposes of inhalation "Formawn," in the compact and convenient form of tablets, is introduced into a simple inhaler consisting of a glass flask and nose-piece. To the flask is added warm water, and the tablet slowly dissolves. As the water cools the flask may be warmed over a stove or spirit-lamp, and the inhaler is again ready for use. We have tested "Formawn" ourselves, and in a number of cases with considerable advantage. In early cases it appears to arrest or modify the inflammatory process, and even when the catarrh is definitely established it affords much alleviation.

### THE MAS-DE-LA-VILLE NON-ALCOHOLIC WINES.

(Ingersoll and Melluish, 6 and 8 Eastcheap, London, E.C.)

These wines are sold in three brands varying in price from 1s. 4d. to 2s. 6d. per quart. We have received samples of two brands—namely, Château Peyron (white label) still and l'Arlesienne (blue label) sparkling. These wines contain no alcohol and consist of pure grape-juice which has been sterilised to prevent the alcoholic fermentation. They contain 26 to 27 ounces of grape-sugar to the gallon. The sparkling varieties are made by introducing carbon-dioxide gas under pressure. They contain no chemical preservative. The wines are clear and of a bright pale sherry colour; their taste is pleasant and not sweet. One sample kept by us opened for a week did not undergo any change. We think that they are pleasant and hygienic beverages, and certainly their freedom from chemical preservatives should be in itself a strong recommendation, indicating, as it does, careful and scientific manufacture.

